# Effect of far infrared therapy on arteriovenous fistula maturation, survival and stenosis in hemodialysis patients, a randomized, controlled clinical trial: the FAITH on fistula trial

**DOI:** 10.1186/s12882-021-02476-x

**Published:** 2021-08-21

**Authors:** K. Lindhard, M. Rix, J. G. Heaf, H. P. Hansen, B. L. Pedersen, B. L. Jensen, D. Hansen

**Affiliations:** 1 Department of Nephrology, Herlev Hospital, Borgmester Ib Juels Vej 1, DK-2730 Herlev, Denmark; 2 Department of Nephrology, Rigshospitalet, Copenhagen, Denmark; 3 Department of Nephrology, University hospital of Zealand, Roskilde, Denmark; 4 Department of Vascular Surgery, Rigshospitalet, Copenhagen, Denmark; 5 Department of cardiovascular and renal research, University Hospital of Southern Denmark, Odense, Denmark

**Keywords:** Arteriovenous fistula, Infrared therapy, AVF patency, AVF maturation, Hemodialysis

## Abstract

**Background:**

An arteriovenous fistula (AVF) is the preferred vascular access for hemodialysis treatment. After creation many of the AVFs will never mature or if functioning will need an intervention within 1 year due to an AVF stenosis. Studies investigating possible therapies that improves the AVF maturation and survival are scarce. Far infrared therapy (FIR) has shown promising results. In minor single centre and industry supported trials FIR has shown improved AVF maturation and survival. There is a need of a randomized multicentre controlled trial to examine the effect of FIR on the AVF maturation and survival and to explore the possible AVF protective mechanism induced by the FIR treatment.

**Methods:**

This investigator initiated, randomized, controlled, open-labeled, multicenter clinical trial will examine the effect of FIR on AVF maturation in patients with a newly created AVF (incident) and AVF patency rate after 1 year of treatment in patients with an existing AVF (prevalent) compared to a control group. The intervention group will receive FIR to the skin above their AVF three times a week for 1 year. The control group will be observed without any treatment. The primary outcome for incident AVFs is the time from surgically creation of the AVF to successful cannulation. The primary outcome for the prevalent AVFs is the difference in number of AVFs without intervention and still functioning in the treatment and control group after 12 months. Furthermore, the acute changes in inflammatory and vasodilating factors during FIR will be explored. Arterial stiffness as a marker of long term AVF patency will also be examined.

**Discussion:**

FIR is a promising new treatment modality that may potentially lead to improved AVF maturation and survival. This randomized controlled open-labelled trial will investigate the effect of FIR and its possible mechanisms.

**Trial registration:**

Clinicaltrialsgov NCT04011072 (7th of July 2019).

## Background

Worldwide, the number of patients with end stage kidney disease in need of hemodialysis therapy increases [1]. In order to receive an efficient hemodialysis treatment, the patient needs a well-functioning and stable vascular access. Currently, there are 3 options: an arteriovenous fistula (AVF), an arteriovenous graft (AVG) and a central venous catheter (CVC) [2, 3]. CVCs are associated with an increased risk of stenosis of the central thoracic vessels, thrombosis in a present AVF, local and systemic infections and death [4–7]. In a multivariate analysis performed by Bray et al., patients with a tunneled CVC almost had a 7 fold increased risk of death from septicemia compared with patients with an AVF [4].

AVGs are associated with increased risk of infection, stenosis in the AVG and loss of access [6, 8, 9]. A study by Almasri et al. [9] showed the need of an intervention (defined as both surgical and endovascular) during 2 years of observation to be 60% in the AVG group compared to 45% in the AVF group. Conclusively, the AVF is the preferred vascular access. However, the AVF is not without complications. Following the creation of an AVF there is a 20–50% risk of maturation failure and consequently, the AVF cannot be used [10, 11]. Furthermore, up to 45–67% of the AVFs will develop a stenosis, that needs an intervention within 1 year [9, 10]. During the period with a malfunctioning AVF, the patient may need a CVC as an alternative vascular access which leads to an increased risk of infection, more hospital days and death [6]. The poor AVF maturation and patency is due to many factors. High age, female sex, anatomic location of the AVF (primarily radio-cephalic), small vein diameter, comorbidity, surgeon experience and perhaps arterial stiffness, are all risk factors that influence the AVF survival [10, 12–15].

Studies investigating possible therapies that improves the AVF maturation and AVF survival are scarce as stated in the vascular access guidelines by European Renal Best Practice and European Society for Vascular Surgery [2, 3]. Furthermore, it is not well established, what causes the neo-intimal hyperplasia in the AVF leading to stenosis and loss of the vascular access [16, 17]. This makes it even more difficult to clarify potential treatments.

Far Infrared therapy (FIR) is a new treatment modality modestly suggested by the European Renal Best Practice to improve AVF maturation and patency [18]. Although suggested, they also call for further studies of FIR, which should be multicentre, preferably blinded and non-industry supported.

FIR is an electromagnetic radiation (heat therapy), that is given directly on the skin above the AVF. The infrared light has in various animal models shown to have a thermal effect, which leads to vasodilatation and angiogenesis [19]. In the clinical setting, FIR is used for wound healing and peripheral ischemia, where it has shown beneficial effect [20]. FIR also promotes a non-thermal effect, where it inhibits vascular endothelial inflammation via induction of anti-inflammatory and vasodilating factors, such as Heme-oxygenase and Nitric oxide [19, 21]. The non-thermal effect is sparsely investigated and understood, especially in hemodialysis patients.

The effect of FIR on the AVF maturation and survival has been explored in a few studies [22–25]. Lin et al. [22, 23] showed a significant improvement in the maturation (90% vs 76% at 3 months) in patients with a newly placed AVF. In patients with a well-functioning AVF with no prior interventions, a lower incidence of AVF malfunction during 12 months of treatment with FIR compared with a control group was found (13% vs. 30%). Lai et al. [24] compared patients in a FIR group with a control group with more than 2 previous interventions on the AVF or AVG. They found a significantly improved unassisted patency rate for the AVGs (16% vs 2%), but not for AVFs (25% vs. 18%). All these studies were randomized, but not blinded. They were performed in one single dialysis department in Taiwan and has not been reproduced or confirmed in other dialysis units and populations.

A study by Choi et al. [25] designed to examine the effect of FIR on cannulation pain and access flow, found no difference in the AVF survival in the FIR treatment group compared to a control group during 12 months of intervention. However, the study was not powered to address this issue. Interestingly, the study showed a significant decrease in the cannulation pain. In conclusion, FIR appears as a promising treatment for AVF maturation and perhaps AVF survival, but the data is inconsistent. There is an urgent need for further investigations of the possible effects of FIR.

The present paper describes the design and rationale of a randomized, controlled, open-labeled multicenter trial of the effect of FIR on AVF maturation and on number of AVFs without intervention still functioning after 1 year. Changes in anti-inflammatory and vasodilating factors during FIR treatment will be explored and arterial stiffness as a marker of AVF maturation and survival will be examined.

## Objectives

1. To evaluate if treatment with FIR for 40 min three times weekly for 1 year will improve the AVF maturation (time to successful cannulation) on newly placed AVFs
2. To evaluate if treatment with FIR for 40 min three times weekly for 1 year will improve the overall AVF patency in prevalent and incident AVFs
3. To evaluate if FIR suppresses inflammatory factors and improve vasodilating factors after one FIR treatment for 40 min
4. To evaluate if arterial stiffness measured by pulse wave velocity can be used as a marker for AVF maturation and patency.

## Methods and design

### Study design and setting

**Fig. 1:**
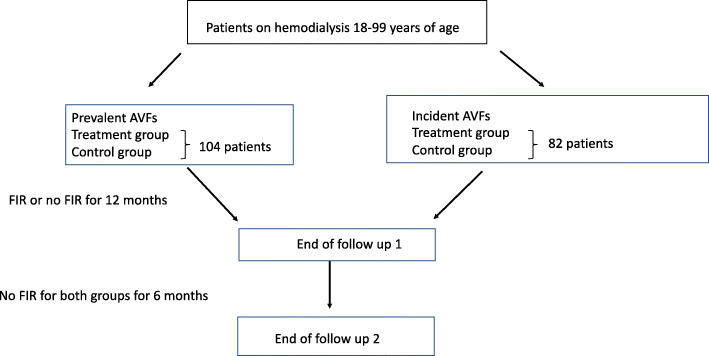
Study design and participants

The FAITH on Fistula trial is a randomized, controlled, open-labeled, multicenter clinical trial. The study design is shown in Fig. 1. The study takes place in a total of 9 dialysis units within the eastern part of Denmark. The dialysis units provide hemodialysis treatment for approximately 1000 hemodialysis patients [26]. All new AVFs will be created by experienced vascular surgeons at 2 hospital departments. Standard criteria’s to assess the arterial and vein adequacy, such as ultrasound, vein diameter > 3 mm, the patients clinical status are used by the vascular surgeons according to the guidelines [2, 3, 27]. Because of the many dialysis units included in the study, the adequate participant enrolment is not difficult to obtain.

The effect of FIR will be examined in two groups of participants; patients on hemodialysis treatment with either prevalent or incident fistulas.

Eligible patients are patients on hemodialysis with a functioning AVF or patients with a CVC, who are scheduled for a new AVF or has an AVF under maturation (maximum 3 weeks old). Inclusion and exclusion criteria are outlined in Table 1. Patient recruitment will be done by the physician in charge at the dialysis unit. All participants are block randomized to FIR or control group 1:1 in blocks of 10. JH will make the sealed randomization envelopes. KL will randomly randomize the patients after written informed consent is given by the patient. The envelopes will be kept in a locked drawer divided in a prevalent AVF group and an incident AVF group and according to dialysis units. Patients with an existing AVF are stratified according to their access flow (< 950 ml/min or ≥ 950 ml/min, the median value for the fistula flow in the largest dialysis unit in Denmark) and number of interventions (0 or ≥ 1 former intervention).

**Table 1:** Study inclusion and exclusion criteria

*Inclusion criteria:*	
Prevalent arteriovenous fistulas	Incident arteriovenous fistula
- 18 years of age or above - Patients on chronic hemodialysis with a functioning arteriovenous fistula	- 18 years of age or above - Patients on chronic hemodialysis with a central venous cathether, who is having a arteriovenous fistula placed - An arteriovenous fistula, that are maximum 3 weeks old
*Exclusion criteria:*	
All groups	
- Not obtained informed consent	
- Non-compliant patients judged by the investigator	
- Patients with both a central venous catheter and an arteriovenous fistula and who uses both for their treatment	
- Combined peritoneal and hemodialysis treatment	
- Planned living donor kidney transplantation	
- Short life expectancy (< 1 year)	
- Patients on hemodialysis < 3 times per week	

### Interventions

**Fig. 2:**
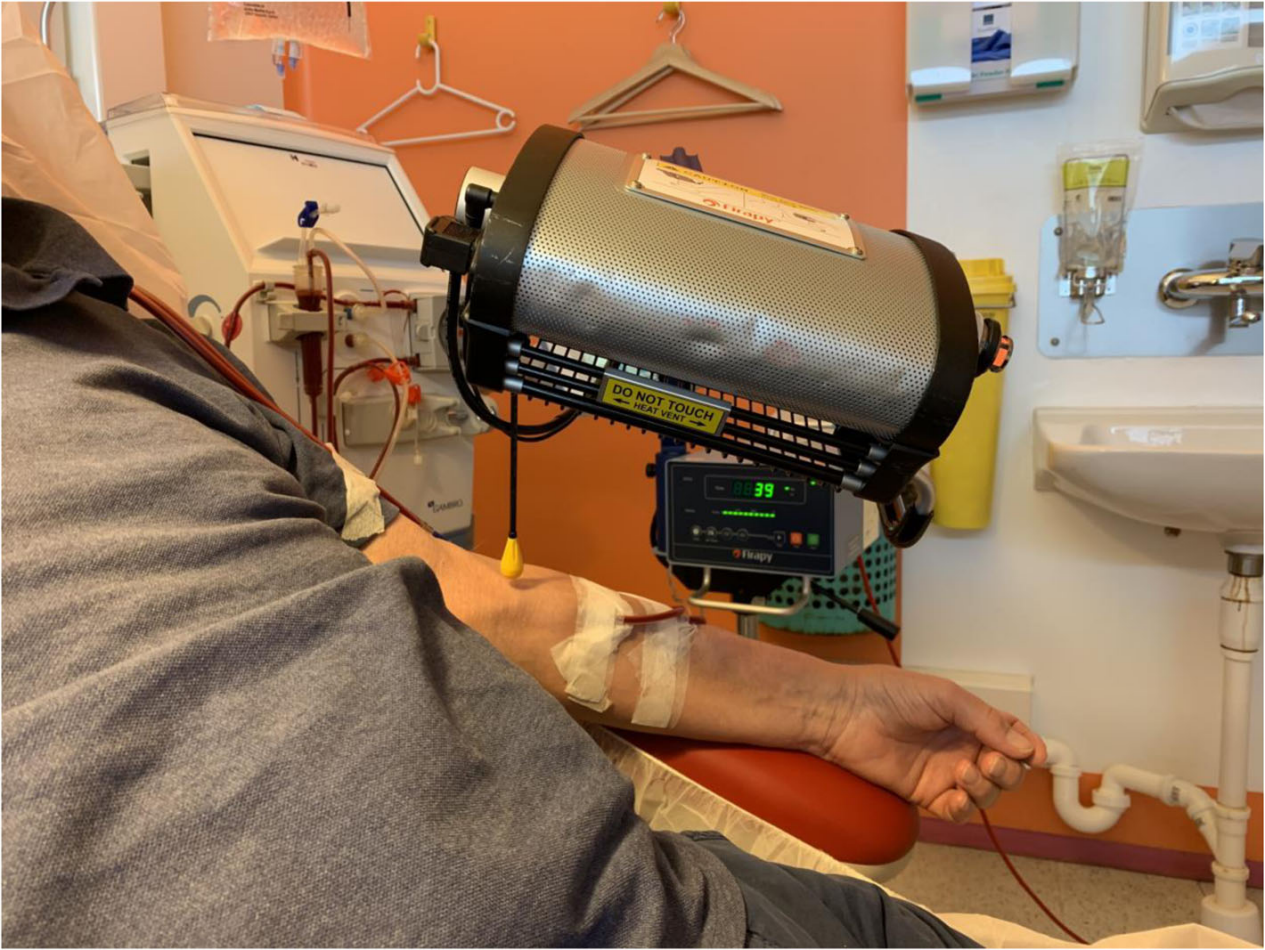
Picture of a enrolled patient receiving FIR treatment on his arteriovenous fistula (informed consent is given)

Ws Far Infrared Therapy Unit, model TY-102F (medical Device Class 11a CE0434, FIRAPY, New Taipei City, Taiwan) is used for the intervention (Fig. 2). The patients in the intervention group will receive FIR above the skin of the AVF for 40 min three times a week for 1 year during a HD treatment. The FIR emitter will be placed 20 cm above the skin surface in the area of the AVF. Every FIR treatment will be registered in the patient chart. The control group will not receive any FIR during the study time, but except from this they will be followed exactly as the intervention group. If the nurses are in doubt of application of the FIR treatment (eg. Hematoma, infection), the primary investigator will be contacted to evaluate the AVF and make the decision.

### Outcomes

The primary endpoint for the prevalent AVFs is the difference in the overall patency rate between the treatment and control group after 12 months. The overall patency rate is defined as the AVFs without intervention and still in function after 1 year of treatment.

The secondary outcomes are the difference after 12 months in the treatment and control group in: access flow, number of abandoned AVF, change of vascular access to CVC or a new AVF, cardiac output, cannulation pain and number of patients with steal syndrome. Additional secondary outcomes are cardiac output and arterial stiffness at baseline as markers for AVF patency. Finally, the acute changes in the inflammatory and vasodilating factors after one FIR treatment will be compared to changes in the control group.

The primary endpoint for the incident AVFs is the time from placement of an AVF to successful cannulation (defined as a successful hemodialysis treatment with two fistula needles placed in the AVF). The secondary outcomes is the difference between the treatment and control group after 12 months in: number of interventions, diameter of the AVF measured by ultrasound, number of abandoned AVF, number of vascular access change (CVC or new AVF), number of primary patency, change in access flow, change in cardiac output, cannulation pain and number of patients with steal syndrome. Additional secondary outcomes are cardiac output and arterial stiffness at baseline as markers for AVF maturation and patency.

### Study visits

Baseline demographic data will be collected from the patient’s hospital file. Subsequent visits will be performed monthly for 12 months. After 12 months the FIR treatment will be ended, but both groups will be followed additionally 6 months. End of study will be at 18 months after baseline. The study visit, procedure and data collection are outlined in Table 2. Participants will be followed until end of study or lost to follow-up, defined by death, change of renal replacement therapy, change of vascular access, if the participant moves away or withdraw their consent. If a patient wish to withdraw their consent, an effort will be made to keep the patient in the study.

**Table 2:**
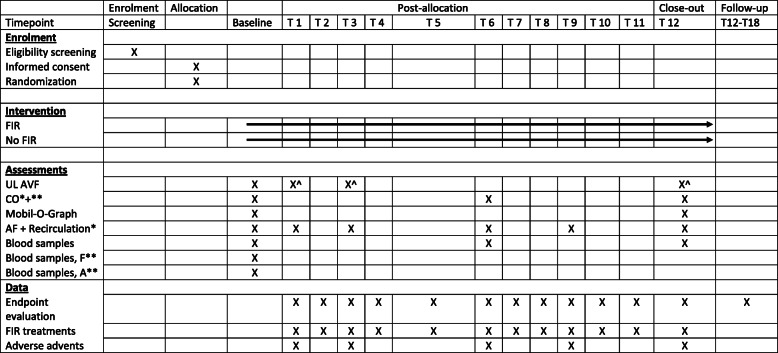
Flow diagram of the study

*Abbreviations: UL* ultrasound, *AVF* arteriovenous fistula, *CO* cardiac output, *AF* access flow, blood samples F blood samples for future research, blood samples a: blood samples acute (for sub study 1)

*: When possible for the new AVF, **: At selected sites only, ^: only for new AVFs

### Sample size and statistics

#### Prevalent AVFs

Al-jaishi et al. and Almasri et al. [9, 10] found a primary patency rate and assisted patency rate for AVFs after 1 year to be 60–69%. Lin et al. found a decline in the incidence of interventions from 30 to 12% after 1 year in the FIR group compared to the control group [23]. We wish to detect an improvement in the overall patency rate and therefore a decline from 35 to 10% in the number of prevalent AVFs with at least one intervention or without function after 1 year of treatment. The 10% is chosen since Lin’s study is from 2007 and we suspect an improved vascular surgery, perhaps with a reduced incidence in interventions. With a power of 80% and a significance of 5% we need 43 patients in each group. With an expected drop out of 20%, the number is 52, resulting in a total of 104 patients for the prevalent AVF group.

Because of the inhomogeneous group in the prevalent AVFs, the patients will be stratified according to previous interventions in the data analysis.

#### Incident AVFs

Hemodialysis patients at the largest dialysis centre in Denmark have a mean of 100 days (SD ± 46) from placement of a new AVF to successful cannulation (defined as a successful HD session with two needles in the AVF). Several guidelines suggest cannulation of a new AVF to be 28–56 days after establishment [2, 3]. Therefore, the minimal relevant difference between the intervention and the control group, which we would like to detect is a decrease from 100 to 70 days before successful cannulation. With a power of 80% and a significance of 5% the number of patients needed for inclusion is 37 patients in each group. With an expected drop out of 20%, the overall number Is 81. Due to equal randomization the total number is 82.

Continuous variables will be expressed as mean ± SD when normally distributed and compared by paired and unpaired *t-test* as appropriate. Non-normally distributed variables will be described as median (range) and compared by Wilcoxon signed rank test and Mann-Whitney U test as appropriate. Categorical variables will be described as numbers and percentages and be analysed by Chi-squared test to compare differences between the intervention and control group.

Survival curves of patency will be calculated by the Kaplan-Meier method and compared by the log rank test. A *p*-value < 0.05 is considered significant.

### Data collection and management

The data collection and timeline are shown in Table 2.

At baseline the following data will be collected:

- Demographics, including renal diagnosis, length of end stage kidney disease, dialysis vintage and previous transplantation.
- Comorbidity.
- Medication
- Pre-study AVF information (previous AVF, anatomic placement of current AVF, AVF vintage, number of interventions on previous and present AVF, cannulation technique, access flow, recirculation, ultrasound of the AVF (cross section in millimetre).
- Pre-study hemodialysis information: hemodialysis/hemodiafiltration, dialysis prescription, Kt/V, anticoagulants during hemodialysis, median blood flow rate, arterial and venous pressure, blood pressure and weight in the latest 3 hemodialysis treatments before baseline, nutritional status defined by the normalized protein catabolic rate.
- For newly created AVFs: acute or permanent CVC, preoperative vein diameter and peroperative accessflow.
- Pre-study side effects: cannulation pain (visual analog score), dialysis side effects, symptoms of steal syndrome.
- Blood samples: haemoglobin, haematocrit, thrombocytes, leucocytes, C-reactive peptide, urea, creatinine, albumin, INR, ionized calcium, phosphate, parathyroid hormone, 25-OH D_2_, fibroblast-growth-factor-23, up-uc-MGP.

Monthly, the following data will be collected:

- Dialysis information: Dialysis prescription, Kt/V, intravenous iron since last visit, anticoagulant during hemodialysis.
- Side effects: Same as baseline and side effects of FIR

The ultrasound examination of the AVF will be performed with Transonic System Inc. HDO3 (Transonic, Ithaca, NY, USA) or Philips Affinity 70 G (Phillips, Andover MA, USA). Access flow and recirculation, will also be measured by Transonic System Inc. HDO3 or Philips Affinity 70 G. Cardiac output will be measured by Transonic System Inc. HCO3. Mobil-O-Graph (IEM, Stolberg, Germany) is used to measure the arterial stiffness.

The AVF will be evaluated at 4 weeks after its creation, whether it is suitable for cannulation. If not, it will be evaluated every week thereafter. The following criteria are used in order to determine if the AVF is ready for cannulation: a diameter of at least 5 mm and no more than 6 mm deep evaluated by ultrasound together with a clinical evaluation of the AVF (e.g. gracile AVF, length, possibility of placing two needles). The evaluation will be done by the same experienced nurse at each dialysis center. Accessflow will be done as soon as possible after successful cannulation. For the prevalent AVFs accessflow will be done according to the study visits.

Data collection will be perfomed by KL. Data entry will be performed and analysed by KL in an approved and secure database, REDCap.

### Sub studies

Two sub studies will be performed:

1. The acute effect of FIR on vasodilating and anti-inflammatory factors in the blood
2. The acute effect of FIR on the blood pressure, access flow, skin temperature, arterial and venous pressure and cardiac output during a dialysis treatment

In study 1, 40 patients will have blood samples collected from their AVF before and after a FIR treatment/no FIR treatment. Another blood sample will be collected in the same time slot from the non-AVF arm. The following factors will be analysed: Serum Amyloid A, Vascular cell adhesion protein, Intercellular adhesion molecule, sE-selectin, Von Willebrand factor, Heme-Oxygenase 1, Nitric oxide products (nitrite, nitrate, asymmetric dimethylarginine), symmetric dimethylarginine, endothelin, Interleukins (IL6, IL8, IL-beta), Tumour necrosis factor alpha, Transforming growth factor beta and Monocyte chemoattractant protein 1. The samples will be kept in a − 80 degrees freezer. The patients will provide another written informed consent for this.

In study 2 the blood pressure will be measured every 5 min before, during and after FIR treatment/no treatment. Furthermore, access flow, recirculation and cardiac output will be measured before, during and after FIR treatment in a subgroup of 10 FIR treated patients and 10 controls.

The baseline data stated previously will be used for the above sub studies. All patients are dialysed on high flux filters. Furthermore, the following data will be collected on the study day: blood pressure, kilograms over dry weight, ultrafiltration volume, arterial and venous pressure, blood flow rate and day of dialysis during the week.

## Monitoring

Previous studies have shown very few side effects of the FIR treatment, such as burning and/or itching sensation. All the data collection will be performed during the patient’s regular hemodialysis treatment, thus there is no extra time for the participant to spend in the department due to the study. Blood samples will be drawn from the dialysis machine, so no extra needling of the patient is needed. Every third month the incidence of side effects will be evaluated. If there is a significant increased incidence in the intervention group (due to FIR) the study will be terminated and the participants will be informed.

Recruitment begun in October 2019. We expect to have completed recruitment in March 2021 for the group with prevalent AVF and in October 2022 for the group with incident AVF with the final data collection in April 2024.

## Discussion

The present study will examine the effect of FIR treatment on AVF maturation and overall patency rate through a randomized, multicenter, open-labelled trial. Furthermore, the acute effects of FIR in terms of changes in anti-inflammatory and vasodilating factors during FIR treatment will be explored and arterial stiffness as a marker for AVF maturation and patency will also be investigated.

After placement of an AVF there is a high risk of maturation failure and need of assisted patency [9, 10, 14]. Approximately 22 to 37% of created AVFs fails and will never be used. By 1 year, 40% of all AVFs fails or have required at least one intervention [10]. Maturation of the AVF depends on several patient related, but also surgically related factors. Factors such as comorbidity (diabetes mellitus, peripheral vascular disease), female sex, length of ESKD, anatomy of the vessel, surveillance after AVF placement, anatomic placement of the AVF and the surgically procedure itself have all been shown to have an influence on the AVF maturation [12, 13, 15, 28, 29].

Studies investigating possible therapies that improves the AVF maturation and survival are scarce. Since the dialysis population is getting older, they present with more comorbidities, and thereby an increased risk of AVF maturation failure. Therefore, there is a need of new interventions that may improve the AVF maturation. FIR is a suggested treatment modality with promising results in the presently available studies [22–24]. There is an urgent need of further studies to explore this treatment. Cannulation of the AVF is by several guidelines suggested to be between 28 to 56 days [2, 3]. However, a huge difference between countries is seen. Early cannulation of the AVF has in studies shown not to reduce the AVF survival [14, 30]. In the largest dialysis center in Zealand, Denmark we found a median time to cannulation of 100 days (SD ± 46) suggesting a poor maturation rate. Lin et al. [22] found a significant improvement in maturation within 1 year (defined in successful cannulation in 8 out of 12 dialysis treatments of a 30 day period) in the FIR treated group compared to the control group (82% vs 60%). The primary endpoint for incident AVFs chosen in our study is therefore time to successful cannulation after AVF placement. Since the FIR treatment is supposed to have a thermal effect increasing blood flow and angiogenesis, we also wish to explore the change of access flow, diameter of the AVF and possible change in cardiac output (perhaps due to the increased access flow in the FIR treatment group). A difference in 1 year patency rate is also examined, since FIR treatment also has shown to decrease the incidence of interventions [22, 23].

After placement of the AVF the patency rates are poor. Al-jaishi et al. and Almasri et al. [9, 10] found a primary patency rate after 1 year of 60–65%. The stenosis in the AVF emerges from an endothelial dysfunction, inflammation and smooth muscle cell proliferation leading to intimal hyperplasia and in the end stenosis [16, 17]. The molecular mechanism that are responsible for the intimal hyperplasia is not well established [16, 28, 31]. Factors, such as Nitric Oxide, Heme-Oxygenase, TNF-alpha and MCP-1 are all factors, that are suggested to be of importance to the AVF maturation and stenosis [16, 31].

There is a need for studies that investigate possible treatments, that may prevent the development of the stenosis and improve the patency of the AVF. Previous studies [18, 32] have explored the effect of different antiplatelet medications, fish oil and prednisolone on the AVF maturation and patency with disappointing results. FIR is suggested by ERBP to be a possible new beneficial treatment for both AVF maturation and patency, but the results of the present studies of the effect of FIR on AVF malfunction is divergent. The studies are also from only one research group and one dialysis unit. Therefore, we designed the present randomized controlled trial to examine the influence of FIR on AVF patency in a Danish population in multiple dialysis units. The primary endpoint for prevalent AVFs is therefore improvement in patency rate in the two groups.

Since peripheral vascular disease is a risk factor for non AVF maturation and AVF survival one can speculate, that arterial stiffness, measured by pulse wave velocity can be used as a prognostic marker for AVF maturation and survival. This has only been explored in a few studies [33–35]. In the studies there were no correlation between arterial stiffness measured by pulse wave velocity and AVF maturation. The follow up time were 6–8 weeks, thus primarily examining the primary failure and maturation of the AVF. Arterial stiffness at baseline as a predictor for long term AVF survival has not previously been explored. This will be clarified in the present study.

The possible mechanisms behind FIR is unraveled. The theory is both a direct vasodilatory effect and a release of various anti-inflammatory and vasodilating factors [20, 21]. FIR has shown positive effects on wound healing and phantom pain in the lower extremities suggesting an increased blood flow and angiogenesis [20]. FIRs effect on a few of the molecular factors involved in the AVF stenosis, have been studied in vitro by Lin et al. [21]. They found a stimulation of heme-oxygenase 1 in endothelial cells (a molecule involved in the relaxation of the smooth muscle cells in the endothelium of the vessels). A decrease of several anti-inflammatory factors, such as tumor necrosis factor and interleukin 8 was also found. There are no studies exploring the acute effect of FIR treatment on these biochemical markers in vivo in a hemodialysis population. This will be explored in the present study.

A large limitation in this study is, that it is not blinded either by investigator, patient or dialysis nurse. Unfortunately, this is not possible due to the size of the FIR emitter and the feeling of the FIR treatment (warm sensation). The majority of the participants enrolled will be of Caucasian origin, and thus the results may not be applicable in other ethnicities. The company, that produces the FIR machine advise, that the FIR treatment can be given during the whole dialysis treatment, except for the last hour (due to risk of prolonged time to hemostasis). In this study the patients will receive the FIR treatment in different time slots during a hemodialysis treatment. If this affects the results is unknown. The optimal timeslot for the FIR treatment during the dialysis session has not previously been investigated. Accessflow on the incident AVFs to determine AVF maturation and time to cannulation is not performed, since this is not standard procedure in the included dialysis units in the study. Other standard criteria’s according to a guideline is used, as described in the Methods and design section.

This project is designed to fill an unmet need regarding methods to investigate factors affecting AVF maturation and patency in an investigator initiated and driven study.

### Potential impact of the trial

All previous studies favours AVF before CVC and AVG, respectively [4–10, 14]. Due to the high risk of stenosis, thrombosis, infection, infection-related mortality and all-cause mortality in patients with AVG and CVC, the need of a well-functioning AVF is of great importance. FIR may be a new treatment modality, which can induce maturation of the AVF and prevent stenosis [22–24], but previous studies are small and single centre with divergent results. The present paper describes the protocol of a randomized, controlled, open-labeled multicenter clinical trial, which will explore if FIR treatment will improve AVF maturation and patency. The acute changes in anti-inflammatory and vasodilating factors will also be explored and this will increase our knowledge of the mechanism of the FIR treatment. Finally, arterial stiffness as a marker for AVF patency will be examined.

If FIR shows to be beneficial on early AVF maturation and AVF patency it will have a positive impact on dialysis patients and their AVF worldwide.

## Data Availability

Not applicable.

## Abbreviations

AVF: arteriovenous fistula
FIR: far infrared therapy
AVG: arteriovenous graft
CVC: central venous catheter
SD: standard deviation
